# Investigating the Mechanism for the Enhanced Oxidation Stability of Microencapsulated Omega-3 Concentrates

**DOI:** 10.3390/md17030143

**Published:** 2019-02-28

**Authors:** Qiuyu Xia, Taiwo O. Akanbi, Bo Wang, Rui Li, Wenrong Yang, Colin J. Barrow

**Affiliations:** 1 Coconuts Research Institute, Chinese Academy of Tropical Agricultural Sciences, Wenchang 571339, China; qiuyu601@outlook.com (Q.X.); liruihn@163.com (R.L.); 2 Centre for Chemistry and Biotechnology, Deakin University, Geelong, VIC 3217, Australia; taiwo.akanbi@deakin.edu.au (T.O.A.); Wenrong.Yang@deakin.edu.au (W.Y.); 3 Nu-Mega Ingredients Pty Ltd., Brisbane, QLD 4113, Australia; BoW@nu-mega.com

**Keywords:** anchovy oil, enzymatic concentration, microencapsulation, oxidative stability, mechanism, concentrates

## Abstract

Enzymatically concentrated anchovy oil (concentrate) is known to be much less stable than unconcentrated anchovy oil. However, we previously showed that concentrate surprisingly forms more stable microcapsules, when produced by complex coacervation, than does unconcentrated anchovy oil. Here we investigate the mechanism of this unexpected stability. We also investigate whether or not incorporation of concentrate can be used as an additive to improve the stability of unconcentrated anchovy oil microcapsules. Results showed that microcap stability increased as the amount of added concentrate increased. Decreased emulsion droplet size, lower positively charged zeta potential, and higher surface hydrophobicity were observed in the oil/water (O/W) emulsion, with the incorporation of concentrate in the oil phase, compared with the unconcentrated anchovy oil O/W emulsion. Both the decreased zeta potential and the increased hydrophobicity of concentrate in the mixed oil phase may improve droplet agglomeration, leading to enhanced oxidative stability of the concentrate-containing microcapsules. Decreased repulsive forces between droplets result in a more compact structure, thicker outer shell, and smoother surface, resulting in enhanced oxidation stability of the concentrate-containing microcapsules.

## 1. Introduction

Omega-3 polyunsaturated fatty acids (PUFAs) are essential fatty acids and have been shown to be associated with important nutritional and biological functions, including inflammation, membrane lipid composition, signal transduction, cellular metabolism, and the regulation of gene expression [1,2], with these properties primarily attributed to the long-chain omega-3 fatty acids eicosapentaenoic acid (EPA, 20:5 n-3) and docosahexaenoic acid (DHA, 22:6 n-3). With increasing awareness of the health benefits of omega-3 PUFAs and the low levels of their dietary intake in many Western countries [3], a large amount of research has been carried out on the development of food delivery systems for these oxidatively unstable oils [4]. This is particularly important since even low levels of omega-3 PUFA oxidation products can produce undesirable odor and cause sensory problems in the fortified products [5,6].

Microencapsulation techniques, with the susceptible compounds as ‘core’ material entrapped within a surrounding ‘wall’ material, can be employed to protect bioactive compounds against adverse reactions such as oxidation [7], nutritional deterioration [8], or volatile loss [9] during their production, storage, and handling [10]. A range of microencapsulation methods have been applied to omega-3 oils, with the most commercially useful being spray-dried emulsions and complex coacervation. Complex coacervation has several advantages over spray-dried emulsions, including higher payload, thicker outer shell, better control of droplet size and particle size, and lower surface oil [11]. Wang et al. (2014) reported significantly enhanced oxidative stability of fish oil based on microencapsulation using complex coacervation [12]. However, the microencapsulation process is costly and so it is important for commercial viability to deliver as high a dose of omega-3 as possible. [5,6,13]. One way to achieve a high omega-3 payload per gram of oil is to use a concentrated omega-3 oil. Therefore, microencapsulating omega-3 acylglycerol concentrates prepared using enzymatic hydrolysis is a useful strategy to increase the EPA and DHA dose per gram of powder, thereby lowering the cost per dose of omega-3. However, omega-3 oil stability decreases significantly as levels of EPA and DHA rise, implying that microencapsulated omega-3 acylglycerol concentrates should be significantly less stable than those that are produced from unconcentrated oils.

We previously produced acylglycerol concentrates by selective partial hydrolysis using *Thermomyces lanuginosus* lipase (TL 100L) [14] and subsequently microencapsulated these concentrates using complex coacervation [12]. Surprisingly, this microencapsulated omega-3 acylglycerol concentrate exhibited enhanced oxidative stability compared to the microencapsulated anchovy oil, even though the omega-3 acylglycerol concentrate itself was much less stable than the native oil [15]. However, the mechanism of this improvement remains unknown. In this study, we investigated possible factors contributing to the enhanced stability of microencapsulated omega-3 acylglycerol concentrate. We also added varying amounts of omega-3 acylglycerol concentrate into unconcentrated refined anchovy oil prior to the microencapsulation and thereby determine if small amounts of added acylglycerol concentrate could stabilize anchovy oil microcapsules. Anchovy oil was used in this study since it contains relatively high levels of both EPA and DHA, and is the major commercial source of both nutritional and pharmaceutical omega-3 products.

## 2. Results 

### 2.1. Oxidative Stability of Anchovy Oil, Anchovy Oil Acylglycerol Concentrate, and Refined Anchovy Oil Microcapsule

**Figure 1:**
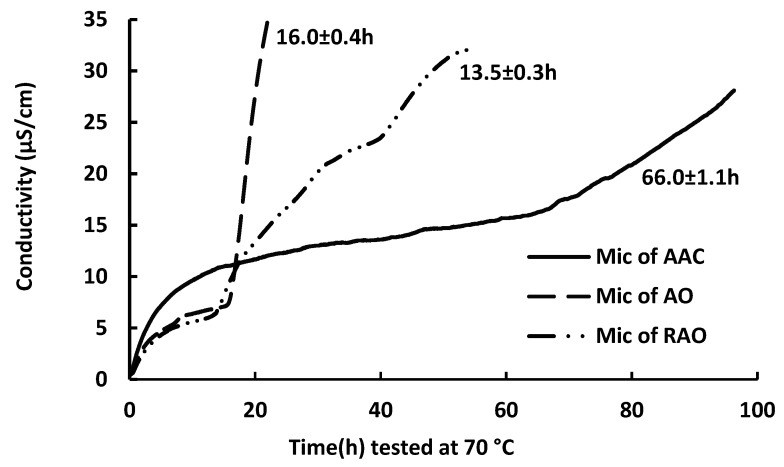

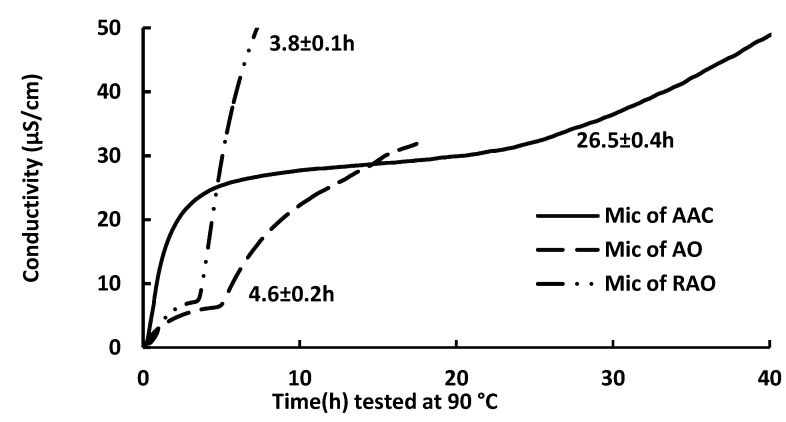
OSI of anchovy acylglycerol concentrate (AAC) microcapsule, anchovy oil (AO) microcapsule, and refined anchovy oil (RAO) microcapsule tested at 70 °C and 90 °C, respectively.

Accelerated oxidation using Rancimat to test lipid oxidative stability is a fast and reliable analytical method [16] with good repeatability [17]. Figure 1 shows the oxidative stability index (OSI) of three different microcapsules (Mic) test using Rancimat at 70 °C and 90 °C, respectively. 

As shown in Figure 1, the OSI of unconcentrated anchovy oil (AO) microcapsule were 16.0 h at 70 °C and 4.6 h at 90 °C, respectively, while the microencapsulated enzymatic anchovy acylglycerol concentrate (AAC) with degree of hydrolysis of approximate 30% exhibited significantly enhanced OSI value, 66.0 h at 70 °C and 26.5 h at 90 °C, respectively. All experiments were carried out in triplicates and the data was replicable.

In order to further study whether this enhancement in the oxidative stability of the microcapsules was as a result of the separation process where the concentrate was extracted from the anchovy oil hydrolysis, the same hydrolysis (lipase blank) and separation process was also carried out on unconcentrated anchovy oil and the separated lipids are referred to below as ‘refined anchovy oil’. The refined anchovy oil (RAO) was microencapsulated in the same encapsulation process and its OSI was found to be slightly lower compared to the unconcentrated anchovy oil microcapsule (13.5 h at 70 °C and 3.8 h at 90 °C, respectively), suggesting the separation process of the lipids did not contribute to the observed enhanced OSI value of the microcapsules.

### 2.2. Effect of Homogenizer Conditions on the OSI of Microcapsule

**Figure 2:**
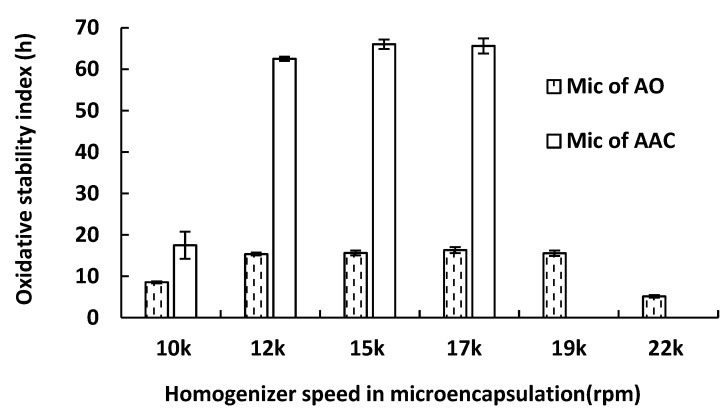
Effect of homogenizer speed on the OSI of concentrate (AAC) microcapsule and unconcentrated anchovy oil (AO) microcapsule.

During microencapsulation, it was observed that the droplet size of the concentrate O/W emulsion was significantly smaller than that of the unconcentrated anchovy oil O/W emulsion (as shown in Figure 6). In order to investigate if the enhanced oxidative stability of the concentrate microcapsule was caused by the decreased size of the O/W emulsion droplets, both unconcentrated anchovy oil and concentrate O/W emulsions were prepared in a homogenization range of 10,000 to 220,000 rpm for 15 min, followed by microencapsulation using complex coacervation under the same conditions. The OSI values of the final dried microcapsules are shown in Figure 2, and show that unconcentrated anchovy oil microcapsule had an OSI value less than 20 h across the homogenization speed range, while the concentrate microcapsules exhibited significantly higher OSI values (*p* < 0.01) than those of the unconcentrated anchovy oil microcapsules at the same homogenization speed. The OSI value of unconcentrated anchovy oil microcapsules prepared using the O/W emulsion homogenized at 22,000 rpm dropped dramatically, possibly due to air entrapped during homogenization. Wang et al. (2015) also reported the entrapment of the air within the microencapsulation system compromised stability, as measured by OSI, for some microcapsule [10]. Interestingly, the OSI value of unconcentrated anchovy oil microcapsules prepared from O/W emulsion homogenized at 22,000 rpm was significantly lower than that of the concentrate microcapsules produced from O/W emulsion homogenized at 15,000 rpm (5 vs. 60 h), even though the average droplet size of the O/W emulsion tested by Zetasizer Nano ZS was similar (both approximately 730 nm). Hence, the enhanced oxidative stability of the microencapsulated concentrate does not appear to be primarily due to the differing O/W emulsion droplet size. 

### 2.3. Oxidative Stability of Anchovy Oil Microcapsules with a Range of Concentrate Content

**Figure 3:**
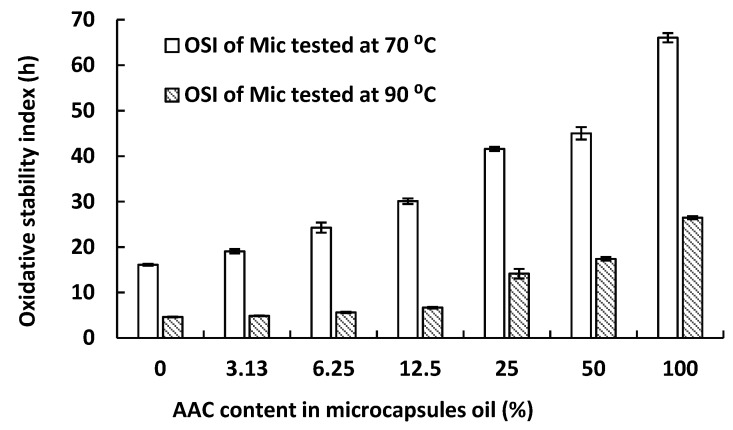
Effect of incorporated concentrate (AAC) on the OSI of microencapsulated anchovy oil.

The impact of added concentrate on the oxidative stability of the microencapsulated mixed oil phase was investigated. Concentrate was mixed with unconcentrated anchovy oil from 0–100% (w/w) (concentrate/mixed oil phase), followed by homogenization and dehydration under the same conditions. The OSI values of the produced microcapsules are shown in Figure 3, which shows that as the level of concentrate increases so does the OSI stability. This indicates that the addition of concentrate can stabilize unconcentrated oil microcapsules, but only in proportion to the amount of concentrate added. That is, the addition of small amounts of concentrate results in only a small amount of stabilization. This result is consistent with droplet size change not being the primary factor impacting microcapsule stability, since the addition of a small amount of concentrate (3.13%) results in the largest decrease in droplet size, but not the largest increase in stability. 

### 2.4. Lipid Class Analysis of the Acylglycerol

**Figure 4:**
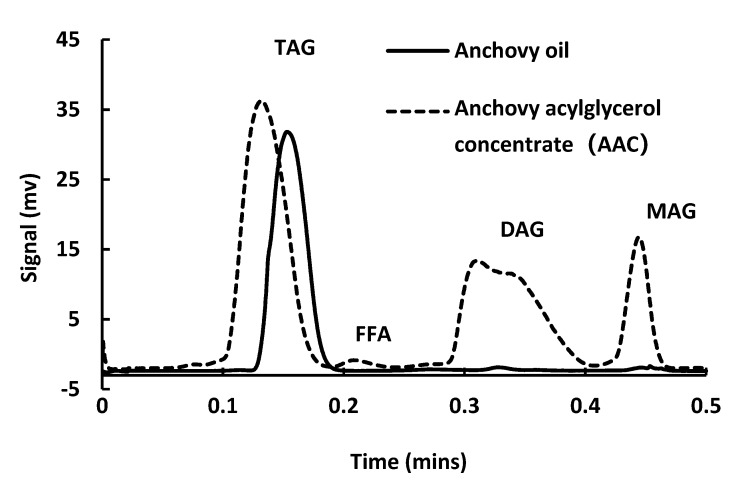
Lipid class analysis of anchovy oil and concentrate (AAC).

The lipid class and fatty acids profiles of unconcentrated anchovy oil and concentrate were compared. Lipid class was analyzed using capillary chromatography (Figure 4), with anchovy oil being 100% triacylglycerol (TAG), while after hydrolysis appreciable levels of monoacylglycerol (MAG), diacylglycerol (DAG), and free fatty acid (FFA) were observed in the oil phase. The TAG, FFA, MAG, and DAG content in the acylglycerol concentrate were 53.7, 1.24, 31.0, and 12.4%, respectively. Most of the FFA in the hydrolysis product was removed during the separation of concentrate. MAG and DAG are effective surfactants and nonionic emulsifiers, which are widely used in food products because of their emulsifying, stabilizing, and conditioning properties [18,19]. Moreover, MAG is also widely used in pharmaceutical products, as binders in tablets, as emollients for transdermal products and in slow-release drugs [20]. Hence, we anticipate that MAG and DAG are embedded within the wall material, facilitating droplet agglomeration during complex coacervation in the cooling step by ‘binding’ the O/W emulsion droplets by hydrophobic forces.

Waraho et al. [21] reported antioxidative activity for MAG and DAG in a soybean oil-in-water emulsion, which may be due to the forming of a liquid crystal physical barrier surrounding the oil phase, decreasing interactions between unsaturated fatty acids and prooxidants or oxygen [22]. In the current research, during homogenization, some MAG and DAG in the oil phase may adsorb at the O/W interface to form a ‘hydrophobic shell’, which provided superior oxidative stability, compared to the unconcentrated anchovy oil microcapsules, which are entrapped by the ‘double complex coacervates shell’. Miyashita, et al. (1997) also reported higher oxidative stability of polyunsaturated monoacylglycerols compared to triacylglycerol in aqueous micelles [23].

### 2.5. Fatty Acid Composition Analysis of the Acylglycerol

**Figure 5:**
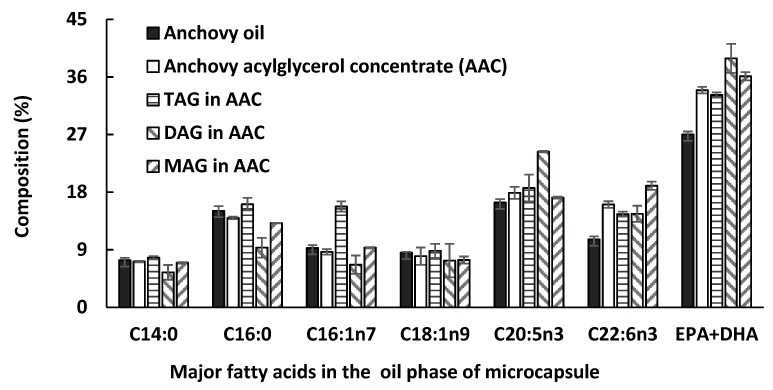
Fatty acid profile for anchovy oil, concentrate, and the separated single acylglycerols (TAG, DAG, and MAG) from concentrate.

Our previous research has shown that the EPA content in the unconcentrated anchovy oil and concentrate were similar, while the DHA content in concentrate was found to be significantly higher [15]. In the current research, the MAG, DAG, and TAG components of concentrate were separated by TLC plate and their fatty acids were analyzed by GC, with results shown in Figure 5. There were some fatty acid differences between MAG, DAG, and TAG, with more EPA found in DAG than TAG and more DHA in MAG than in TAG. The total content of EPA and DHA was 38.9% in the DAG form, and 36.1% in MAG, which were both higher than in TAG (33.2%). This is consistent with the lipase preferentially hydrolyzing shorter chain fatty acids and saturates. Omega-3 containing MAG and DAG may have improved bioavailability compared with TAG [24]. 

### 2.6. Effect of the Concentrate Incorporation on the Interfacial and Emulsion Characteristics of Gelatin-Stabilised Oil-in-Water Emulsions

**Figure 6:**
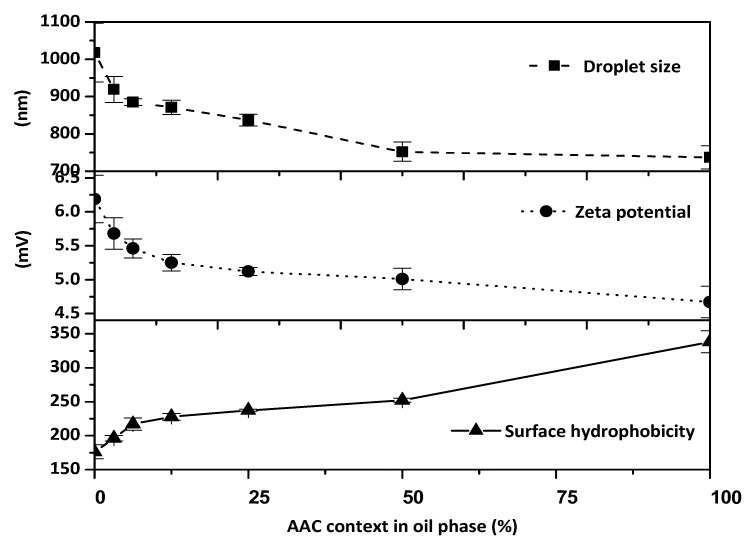
Interfacial and emulsifying properties analysis of oil-gelatin emulsion using anchovy oil incorporated with different amounts of concentrate.

Results from the characterization of the O/W emulsion during microencapsulation, stabilized by gelatin before complex coacervation—including emulsion droplet size, zeta-potential, and surface hydrophobicity—are shown in Figure 6. At the same homogenization speed, the O/W emulsion exhibited a significantly smaller droplet size increasing concentrate content in the mixed oil phase, from 1018 ± 79 nm in anchovy oil emulsion to 737 ± 31 nm in concentrate emulsion. Unlike TAG, MAG and DAG are more effective surfactants and emulsifiers. Bornscheuer [18] and Feltes et al. [19] reported surface tension reduction when MAG and DAG were adsorbed at the O/W interface during homogenization, which improved emulsification and their adsorption at the oil/water interface [18,19]. Therefore, the presence of MAG and DAG can result in finer emulsions with smaller and more uniform droplet sizes during homogenization [25,26]. 

Zeta-potential of the O/W emulsion decreased with an increase in the concentrate content in the mixed oil phase, from 6.19 ± 0.35 mV in anchovy oil emulsion to 4.67 ± 0.43 mV in concentrate emulsion, probably because of less adsorbed positively charged gelatin at the O/W interface and a smaller O/W droplet size. Recent research indicates that surfactants and polymers will impact the zeta potential significantly in fine particle colloidal suspensions, and accelerate particle aggregation decreasing the absolute value of the zeta potential [27]. Zeta potential is a critical factor for the stability of colloidal dispersions and has been widely used to quantify the magnitude of electric charge distribution that surrounds particles and characterizes the stability of colloidal dispersions [28]. Colloids are electrically stabilized when they have high zeta potential, and when the magnitude of the zeta potential decreases, attractive forces may exceed this repulsion causing the dispersion to break and flocculate [29]. When the zeta potential is between ±30 to ±10 the colloidal dispersions will be incipiently unstable. When the Zeta potential decreases from 0 to ±5, the colloidal dispersions will rapidly coagulate or flocculate [30,31]. 

However, the surface hydrophobicity of the O/W emulsion droplets significantly increased with an increase in the concentrate content in the mixed oil phase, from 176.11 ± 10.15 in anchovy oil emulsion, increasing to 338.38 ± 15.98 in concentrate emulsion. This decreased O/W emulsion droplet resulted in more overall droplet surface area and therefore a lower ratio of gelatin to hydrophobic droplet surface area, which may explain the change in surface hydrophobicity.

### 2.7. Physicochemical Properties of Various Microcapsules Using Anchovy Oil Incorporated with Various Amounts of Concentrate

The physicochemical properties of the prepared microcapsules were investigated. The surface oil content, total oil, encapsulation efficiency, and microcapsule droplet size are shown in Table 1. The high payload (>50%) and extremely high encapsulation efficiency (>95%) of the microcapsules in this study are consistent with our previous work [12,15]. Surface oil content increased with an increase of concentrate in the mixed oil phase, together with a decrease in encapsulation efficiency, perhaps due to higher levels of FFA in the concentrate. This FFA content might also contribute to the increased conductivity in the concentrate microcapsules at the beginning of the heating in the accelerated oxidation test, as shown in Figure 1.

## 3. Discussion

**Figure 7:**
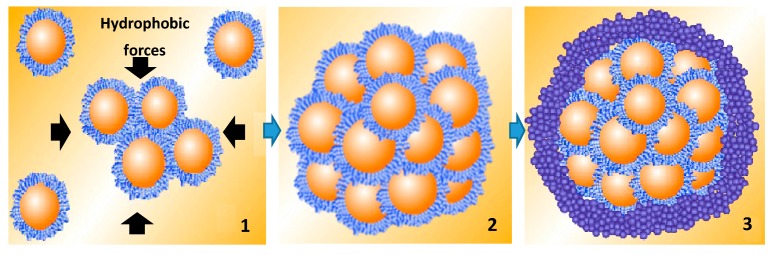
Key process of complex coacervation to form microcapsule during the cooling step.

Both the decreased zeta potential and the increased hydrophobicity of concentrate in the mixed oil phase may improve droplet agglomeration leading to enhanced oxidative stability of the microcapsules. Droplet size is an important factor, but since a small amount of concentrate (3.13%) has the largest impact on droplet size (Table 1) but not on stability (Figure 3) other factors play an important role in stability. Zeta potential and increasing hydrophobicity correlate with improved stability and are important factors in strengthening the interactions between particles, leading to a more compact structure and greater oxidative stability. The complex coacervation microencapsulation process during the cooling step is shown in Figure 7. Step 1 and Step 2 show the emulsion droplets agglomeration during complex coacervation, with the arrows in Step 1 representing the surrounding hydrophobic forces. Step 3 shows the outer shell formation. O/W emulsion droplet agglomeration occurred because of the formation of the ‘free complex coacervates’, which was in the surrounding aqueous phase rather than at O/W interface. During the cooling step, these free coacervates adsorb onto the surface of the agglomerated droplets to form an outer shell, due to hydrophobic forces. In the presence of concentrate in the oil phase, the microcapsules tended to have a more compact structure due to their decreased droplet size, decreased zeta-potential values (i.e., weaker repulsive forces) and increased surface hydrophobicity (i.e., stronger attractive hydrophobic forces) in the O/W emulsion. In addition, the outer shell in microcapsules containing concentrate appear to be thicker and smoother, probably as a result of the more compact internal structure.

## 4. Materials and Methods 

### 4.1. Materials

Anchovy oil was provided by DSM Nutritional Products (Dartmouth, Nova Scotia, Canada) and stored at 4 °C before use. *Thermomyces lanuginosus* lipase (TL 100L) was obtained from Novozymes Australia Pty. Ltd. (North Rocks, NSW, Australia). Antioxidant Duralox Blend AN 110XT were provided by Kalsec Incorporation (Kalamazoo, MI, USA). Transglutaminase (Activa^®^ KS–LS) was purchased from Ajinomoto (Tokyo, Japan) and stored at 4 °C before use. All other chemicals used were analytical grade and purchased from Sigma-Aldrich Australia (Sydney, NSW, Australia).

### 4.2. Preparation of Anchovy Oil Acylglycerol Concentrate 

Enzymatic anchovy acylglycerol concentrate (concentrate) was prepared according to a previous reported method [14,15] with minor modifications: 100 g anchovy oil was mixed with the lipase TL 100 L at the dosage of 2000 U/g oil, followed by the addition of 0.1 M pH7.5 phosphate buffer at the oil/buffer ratio of 1.8 mL/g. The mixture was flushed with nitrogen and incubated under the agitation at 300 rpm in the phosphate buffer at 40 °C for 3 h, then KOH solution (0.5 M in 30% ethanol) was used to neutralize the free fatty acids (FFA) released during the hydrolysis [32] and the acylglycerol portion was separated using diethyl ether and then heptane. The collected concentrate was kept in glass bottles in the presence of nitrogen at 4 °C until used.

### 4.3. Preparation of Oil-in-Water Emulsion 

Twenty g of oil was mixed with 150 g (8%, w/w) gelatin dispersion and this mixture was stirred using a mechanical stirrer at 1200 rpm for 5 min. The mixture was then emulsified at 15,000 rpm for 15 min using a homogenizer (Unidrive 1000, CAT Scientific, Paso Robles, CA, USA) to prepare the oil-in-water (O/W) emulsion for microencapsulation. Then 2 g O/W emulsions were withdrawn and diluted 20 times using Milli-Q water to avoid their gelation, the emulsion was stored at 4 °C as the stock emulsions before their droplet size distribution, zeta-potential, and surface hydrophobicity determination.

### 4.4. Microencapsulation of Oil Phases Using Complex Coacervation

Microcapsules of anchovy oil, anchovy oil concentrate, and their mixture were prepared using gelatin-sodium hexametaphosphate complex coacervates as shell material based on our previous study [12,15]. Antioxidant Duralox Blend AN 110 XT was incorporated to the oil phase at 0.4% (w/w) prior to microencapsulation to avoid the oxidation of the omega-3 oils during the processing.

### 4.5. Accelerated Oxidative Stability Test of Microcapsules

Accelerated oxidation tests of microcapsule were carried out using a Rancimat (Model 743, Herison, Switzerland) following a previously described method [12]. 2 g microcapsules were heated at 70 °C or 90 °C under a purified air, at a flow rate of 10 L/h. The induction times of the tested samples were recorded and used as their oxidative stability index (OSI).

### 4.6. Separation of Lipid Class by TLC

Lipid class was separated by TLC. 500 mg of oil sample was suspended in 5 mL of heptane and carefully spotted onto thin layer chromatographic plates (TLC; 20 × 20 cm) (Merck). Plates were developed in hexane/diethyl ether/acetic acid (60:17:0.2) for 22 min and spots were visualized after spraying the plate with 60% H_2_SO_4_ and dried at 120 °C for 30 min. Once the lipid classes have been identified using standards, spot representing each lipid class was scraped off after being visualized under UV, resuspended in methanol for 12 h and then further analyzed by Iatroscan and GC. Each lipid class was identified using the SIC-480 II software for multiple chromatogram processing. 

### 4.7. Analysis of Lipid Class and Fatty Acid Profile 

The lipid classes of the native anchovy oil and concentrate were analyzed by capillary chromatography with flame ionization detector (Iatroscan MK5, Iatron Laboratories Inc., Tokyo, Japan) based on our established method [14,33]. The major fatty acid profile analysis using a gas chromatograph (6890 model, Agilent Technologies, Santa Clara, CA, USA) was performed using a previously reported method [15,34]. 

### 4.8. Measurement of Emulsion Average Droplet Size and Zeta Potential

The stock emulsions were further diluted 5-fold and the average droplet size and zeta potential values of the O/W emulsion droplets were analyzed using a NanoZS Zetasizer (Malvern Instruments Ltd., Worcestershire, UK). 

### 4.9. Measurement of Surface Hydrophobicity

The surface hydrophobicity of the emulsion was measured according to previously methods with some modifications [35,36]. Stock O/W emulsions were double diluted with Milli-Q water to 320, 640, 1280, 2560, 5120, and 10240 times, and then the droplet surface hydrophobicity was measured using 1-anilinno-napthalene sulphonate (ANS) as a hydrophobic probe. 20 μL of ANS (4.0 mM in 0.1 M phosphate buffer, pH 7.0) was added to 4 mL of diluted O/W emulsions and mixed well, followed by the incubation in darkness for 30 min at room temperature. The fluorescence intensity (FI) of the mixtures was measured using Cary Eclipse spectrofluorimeter (Varian Australia, Sydney, Australia). FI for ANS solution alone was determined by using the same amount of ANS in Milli-Q water and the relative fluorescence intensity (RFI) for each concentration was calculated using Equation (1) given below [37]:

(1) $$RFI=\frac{F_{s}-F_{0}}{F_{0}}$$

where *F_s_* and *F*_0_ are fluorescence intensity of protein-ANS conjugate and ANS alone, respectively. The surface hydrophobicity was expressed as the initial slope of the plot of RFI versus protein concentration (mg/mL).

### 4.10. Physicochemical Properties of the Microcapsules

The physical properties of microcapsules, including moisture content, surface oil content, total oil content, encapsulation efficiency (EE), payload (PL), and encapsulation yield (EY), were determined based on reported methods [12].

### 4.11. Particle Size Distribution of Microcapsules

The particle size distribution of the final microcapsule powder was determined using a Malvern Mastersizer 2000 (Malvern Instruments Ltd., Worcestershire, UK). The volume surface average diameter, *d*(3, 2) (µm), volume weighted average diameter, *d*(4, 3) (µm) and dispersion index (Span) were calculated using Equations (2), (3), and (4), respectively.

(2) $$d\left(3,2\right)=\frac{\sum n_{i}{d_{i}}^{3}}{\sum n_{i}{d_{i}}^{2}}$$

(3) $$d\left(4,3\right)=\frac{\sum n_{i}{d_{i}}^{4}}{\sum n_{i}{d_{i}}^{3}}$$

(4) $$Span=\frac{d\left(0.9\right)-d\left(0.1\right)}{d(0.5)}$$

where, *n_i_* is the number of the droplets with the same diameter; *d_i_* is the droplet size. The *d*(0.1), *d*(0.5) and *d*(0.9) are the average droplet size values corresponding to the cumulative distribution at 10, 50, and 90%, respectively.

### 4.12. Statistical Analysis

All the experiments were carried out in triplicates and the SPSS statistical package (IBM SPSS statistics 21, Foster City, CA, USA) was used for the analysis of variance (ANOVA) to test the significant difference between the mean values.

## 5. Conclusions

In the current study, we investigated the stabilizing effect of MAG and DAG on anchovy oil containing microcapsules. Possible reasons for the enhanced oxidative stability of concentrate microcapsules were investigated. The concentrate separation process and the O/W emulsion droplet size under various homogenization speeds were found not to be the most significant factors in stability enhancement. The involvement of concentrate during homogenization, specifically, the possible embedment of the DAG and MAG in the complex coacervate shell, may be partially responsible for the significantly improved OSI of the concentrate microcapsules. Moreover, the smaller size, lowered zeta-potential and higher hydrophobicity of the O/W droplets with high levels of concentrate content in the mixed oil phase may improve droplet agglomeration packing, leading to enhanced oxidative stability of the microcapsules. This more density compacted internal structure appears to also enable the formation of a thicker outer shell, further improving microcapsule stability.

## Figures and Tables

**Table 1:** Physical properties of various microcapsules using anchovy oil incorporated with differing amounts of concentrate.

Concentrate Context in the Mixed Oil Phase	Surface Oil (%)	Total Oil (%)	Encapsulation Efficiency (%)	Microcapsule Droplet Size		
				D [4,3] (µm)	D [3,2] (µm)	Span
Unconcentrated anchovy oil	0.82 ± 0.27a	54.83 ± 1.17	98.51 ± 0.51	96.00 ± 8.80d	63.99 ± 2.28d	1.78 ± 0.39
3.13% Concentrate	1.91 ± 0.22b	53.02 ± 0.84	96.40 ± 0.39	69.29 ± 7.25c	40.20 ± 3.87c	1.73 ± 0.05
6.25% Concentrate	1.64 ± 0.23b	53.92 ± 0.57	96.97 ± 0.41	62.83 ± 2.91c	40.30 ± 6.24c	1.52 ± 0.01
12.50% Concentrate	2.26 ± 0.19c	53.21 ± 0.94	95.76 ± 0.42	57.41 ± 1.85b	32.25 ± 4.48b	1.47 ± 0.10
25.00% Concentrate	2.16 ± 0.25c	52.42 ± 0.96	95.89 ± 4.14	52.58 ± 1.24a	35.67 ± 3.16b	1.63 ± 0.01
50.00% Concentrate	2.30 ± 0.54c	53.10 ± 1.16	95.67 ± 0.91	51.77 ± 3.72a	30.94 ± 3.24a	1.55 ± 0.01
Concentrate	2.59 ± 0.57c	53.34 ± 0.81	95.12 ± 1.09	49.34 ± 4.67a	28.86 ± 1.94a	1.85 ± 0.06

The different small letters on OSI values within the same column indicate significant difference by Duncan’s test (*p* < 0.05).

## References

[1] Jacobsen C., Let M.B., Nielsen N.S., Meyer A.S. (2008). Antioxidant strategies for preventing oxidative flavour deterioration of foods enriched with n-3 polyunsaturated lipids: A comparative evaluation. Trends Food Sci. Technol..

[2] Arab-Tehrany E., Jacquot M., Gaiani C., Imran M., Desobry S., Linder M. (2012). Beneficial effects and oxidative stability of omega-3 long-chain polyunsaturated fatty acids. Trends Food Sci. Technol..

[3] Calder P.C., Yaqoob P. (2009). Omega-3 polyunsaturated fatty acids and human health outcomes. Biofactors.

[4] Drusch S., Serfert Y., Scampicchio M., Schmidt-Hansberg B., Schwarz K. (2007). Impact of physicochemical characteristics on the oxidative stability of fish oil microencapsulated by spray-drying. J. Agric. Food Chem..

[5] Barrow C.J., Nolan C., Jin Y. (2007). Stabilization of highly unsaturated fatty acids and delivery into foods. Lipid Technol..

[6] Halvorsen B.L., Blomhoff R. (2011). Determination of lipid oxidation products in vegetable oils and marine omega-3 supplements. Food Nutr. Res..

[7] Hogan S.A., O’Riordan E.D., O’Sullivan M. (2003). Microencapsulation and oxidative stability of spray-dried fish oil emulsions. J. Microencapsul..

[8] Kagami Y., Sugimura S., Fujishima N., Matsuda K., Kometani T., Matsumura Y. (2003). Oxidative Stability, Structure, and Physical Characteristics of Microcapsules Formed by Spray Drying of Fish Oil with Protein and Dextrin Wall Materials. J. Food Sci..

[9] Kim Y.D., Morr C.V. (1996). Microencapsulation properties of gum arabic and several food proteins: Spray-dried orange oil emulsion particles. J. Agric. Food Chem..

[10] Wang B., Vongsvivut J., Adhikari B., Barrow C.J. (2015). Microencapsulation of tuna oil fortified with the multiple lipophilic ingredients vitamins A, D3, E, K2, curcumin and coenzyme Q10. J. Funct. Foods.

[11] Eratte D., Dowling K., Barrow C.J., Adhikari B. (2018). Recent advances in the microencapsulation of omega-3 oil and probiotic bacteria through complex coacervation: A review. Trends Food Sci. Technol..

[12] Wang B., Adhikari B., Barrow C.J. (2014). Optimisation of the microencapsulation of tuna oil in gelatin–sodium hexametaphosphate using complex coacervation. Food Chem..

[13] Kralovec J.A., Zhang S., Zhang W., Barrow C.J. (2012). A review of the progress in enzymatic concentration and microencapsulation of omega-3 rich oil from fish and microbial sources. Food Chem..

[14] Akanbi T.O., Adcock J.L., Barrow C.J. (2013). Selective concentration of EPA and DHA using Thermomyces lanuginosus lipase is due to fatty acid selectivity and not regioselectivity. Food Chem..

[15] Xia Q., Wang B., Akanbi T.O., Li R., Yang W., Adhikari B., Barrow C.J. (2017). Microencapsulation of lipase produced omega-3 concentrates resulted in complex coacervates with unexpectedly high oxidative stability. J. Funct. Foods.

[16] Farhoosh R. (2007). The effect of operational parameters of the Rancimat method on the determination of the oxidative stability measures and shelf-life prediction of soybean oil. JAOCS J. Am. Oil Chem. Soc..

[17] Kaushik P., Dowling K., Barrow C.J., Adhikari B. (2015). Microencapsulation of omega-3 fatty acids: A review of microencapsulation and characterization methods. J. Funct. Foods.

[18] Bornscheuer U.T. (1995). Lipase-catalyzed syntheses of monoacylglycerols. Enzym. Microb. Technol..

[19] Feltes M.M.C., de Oliveira D., Block J.M., Ninow J.L. (2013). The production, benefits, and applications of monoacylglycerols and diacylglycerols of nutritional interest. Food Bioprocess Technol..

[20] Damstrup M.L., Jensen T., Sparsø F.V., Kiil S.Z., Jensen A.D., Xu X. (2005). Solvent optimization for efficient enzymatic monoacylglycerol production based on a glycerolysis reaction. J. Am. Oil Chem. Soc..

[21] Waraho T., Cardenia V., Nishino Y., Seneviratne K.N., Rodriguez-Estrada M.T., McClements D.J., Decker E.A. (2012). Antioxidant effects of mono- and diacylglycerols in non-stripped and stripped soybean oil-in-water emulsions. Food Res. Int..

[22] Da Pieve S., Calligaris S., Nicoli M.C., Marangoni A.G. (2010). Shear nanostructuring of monoglyceride organogels. Food Biophys..

[23] Miyashita K., Hirano S., Itabashi Y., Ota T., Nishikawa M., Nakayama S. (1997). Oxidative Stability of Polyunsaturated Monoacylglycerol and Triacylglycerol in Aqueous Micelles. J. Oleo Sci..

[24] Solaesa Á.G., Sanz M.T., Falkeborg M., Beltrán S., Guo Z. (2016). Production and concentration of monoacylglycerols rich in omega-3 polyunsaturated fatty acids by enzymatic glycerolysis and molecular distillation. Food Chem..

[25] Choi E., Cho Y.I., Lorsch H.G. (1991). Effects of emulsifier on particle size of a phase change material in a mixture with water. Int. Commun. Heat Mass Transf..

[26] Chung C., Sher A., Rousset P., Decker E.A., McClements D.J. (2017). Formulation of food emulsions using natural emulsifiers: Utilization of quillaja saponin and soy lecithin to fabricate liquid coffee whiteners. J. Food Eng..

[27] Guo Y., Zhao Y., Wang S., Jiang C., Zhang J. (2018). Relationship between the zeta potential and the chemical agglomeration efficiency of fine particles in flue gas during coal combustion. Fuel.

[28] Kirby B.J. (2010). Micro- and Nanoscale Fluid Mechanics: Transport in Microfluidic Devices.

[29] Hanaor D., Michelazzi M., Leonelli C., Sorrell C.C. (2012). The effects of carboxylic acids on the aqueous dispersion and electrophoretic deposition of ZrO2. J. Eur. Ceram. Soc..

[30] Wu Z., Wu J., Zhang R., Yuan S., Lu Q., Yu Y. (2018). Colloid properties of hydrophobic modified alginate: Surface tension, ζ-potential, viscosity and emulsification. Carbohydr. Polym..

[31] O’Brien R.W., Midmore B.R., Lamb A., Hunter R.J. (1990). Electroacoustic studies of moderately concentrated colloidal suspensions. Faraday Discuss. Chem. Soc..

[32] Liu Y., Jin Q., Shan L., Liu Y., Shen W., Wang X. (2008). The effect of ultrasound on lipase-catalyzed hydrolysis of soy oil in solvent-free system. Ultrason. Sonochem..

[33] Akanbi T.O., Barrow C.J., Byrne N. (2012). Increased hydrolysis by Thermomyces lanuginosus lipase for omega-3 fatty acids in the presence of a protic ionic liquid. Catal. Sci. Technol..

[34] Akanbi T.O., Sinclair A.J., Barrow C.J. (2014). Pancreatic lipase selectively hydrolyses DPA over EPA and DHA due to location of double bonds in the fatty acid rather than regioselectivity. Food Chem..

[35] Liu H., Wang B., Barrow C.J., Adhikari B. (2014). Relating the variation of secondary structure of gelatin at fish oil-water interface to adsorption kinetics, dynamic interfacial tension and emulsion stability. Food Chem..

[36] Kato A., Nakai S. (1980). Hydrophobicity determined by a fluorescence probe method and its correlation with surface properties of proteins. Biochim. Biophys. Acta.

[37] Chaudhuri T.K., Das K.P., Sinha N.K. (1993). Surface hydrophobicity of a low molecular weight basic trypsin subtilisin inhibitor from marine turtle eggwhite. J. Biochem..

